# Gene expression analyses of immune responses in Atlantic salmon during early stages of infection by salmon louse (*Lepeophtheirus salmonis*) revealed bi-phasic responses coinciding with the copepod-chalimus transition

**DOI:** 10.1186/1471-2164-12-141

**Published:** 2011-03-07

**Authors:** Tariku Markos Tadiso, Aleksei Krasnov, Stanko Skugor, Sergey Afanasyev, Ivar Hordvik, Frank Nilsen

**Affiliations:** 1 Department of Biology, University of Bergen, Thormølhensgate 55, N-5020 Bergen, Norway; 2 Nofima, Norwegian Institute of Food, Fisheries and Aquaculture Research, P.O. Box 5010, Ås NO-1430, Norway; 3 Sechenov Institute of Evolutionary Physiology and Biochemistry, M. Toreza av. 44, St Petersburg, 194223, Russia

## Abstract

**Background:**

The salmon louse (*Lepeophtheirus salmonis *Krøyer), an ectoparasitic copepod with a complex life cycle causes significant losses in salmon aquaculture. Pesticide treatments against the parasite raise environmental concerns and their efficacy is gradually decreasing. Improvement of fish resistance to lice, through biological control methods, needs better understanding of the protective mechanisms. We used a 21 k oligonucleotide microarray and RT-qPCR to examine the time-course of immune gene expression changes in salmon skin, spleen, and head kidney during the first 15 days after challenge, which encompassed the copepod and chalimus stages of lice development.

**Results:**

Large scale and highly complex transcriptome responses were found already one day after infection (dpi). Many genes showed bi-phasic expression profiles with abrupt changes between 5 and 10 dpi (the copepod-chalimus transitions); the greatest fluctuations (up- and down-regulation) were seen in a large group of secretory splenic proteases with unknown roles. Rapid sensing was witnessed with induction of genes involved in innate immunity including lectins and enzymes of eicosanoid metabolism in skin and acute phase proteins in spleen. Transient (1-5 dpi) increase of T-cell receptor alpha, CD4-1, and possible regulators of lymphocyte differentiation suggested recruitment of T-cells of unidentified lineage to the skin. After 5 dpi the magnitude of transcriptomic responses decreased markedly in skin. Up-regulation of matrix metalloproteinases in all studied organs suggested establishment of a chronic inflammatory status. Up-regulation of putative lymphocyte G0/G1 switch proteins in spleen at 5 dpi, immunoglobulins at 15 dpi; and increase of IgM and IgT transcripts in skin indicated an onset of adaptive humoral immune responses, whereas MHCI appeared to be down-regulated.

**Conclusions:**

Atlantic salmon develops rapid local and systemic reactions to *L. salmonis*, which, however, do not result in substantial level of protection. The dramatic changes observed after 5 dpi can be associated with metamorphosis of copepod, immune modulation by the parasite, or transition from innate to adaptive immune responses.

## Background

The salmon louse (*Lepeophtheirus salmonis *Krøyer) is a widespread disease-causing marine ectoparasitic copepod infecting wild and farmed salmonids. The development of *L. salmonis *encompasses ten stages: two nauplii, a copepodid, four chalimus, two pre-adult, and an adult stage [1]. The nauplii hatch directly from egg-strings attached to the female lice. The two nauplii stages and the copepodid are free-living larvae that utilize yolk and other components provided maternally. The copepodid is the infectious stage of *L. salmonis*; its ability to settle and to recognize a relevant host is of critical importance for the parasite. We have observed that *L. salmonis *copepodids use 7-11 days (at 9.3°C) before they all have completed the molt to chalimus I. The four chalimus stages are physically attached to the host by a frontal filament. Even though an increase in virulence by *L. salmonis *has been observed as the parasite reaches the pre-adult stages [2], the chalimus stage can also account for smolt mortalities (e.g. in small pink salmon [3]). Lice damage fish by feeding on their mucus, skin, and blood and the wounds increase the risk of secondary infections. At present, *L. salmonis *is recognized as one of the major problems in salmon aquaculture in Norway, UK, USA, and Canada; whereas in Chile, a *Caligus *species (*C. rogercresseyi*) gives similar problems. The annual global loss due to sea lice in salmonid aquaculture is estimated to be more than 300 million USD [4]. Moreover, lice originating from farmed salmon may cause infections and mortality on wild salmonids [4,5].

*L. salmonis *is controlled mainly by pesticides and at present only a few types are available, emamectin benzoate being the most commonly used [6]. However, increasing concerns about development of pesticide resistance, occurrence of treatment failures, and undesirable environmental impacts raise questions about the future of this strategy. The need for new methods of parasite control is fully recognized by the industry, authorities and society. At this time multiple studies assess improvement of salmon resistance to lice with an aid of selective breeding, special feeds and immune stimulants. The possibility of immunization and vaccination against *L. salmonis *infection is discussed [7,8]. However, protective antibody responses following repeated challenge are weak. Better understanding of acquired immune responses is essential for vaccine development. However, data on factors related to adaptive immunity are lacking in this host-parasite system [7,9]. Development of biological methods of protection needs better understanding of mechanisms underlying resistance to lice. The ability to suppress and reject parasites shortly after infection can be associated with innate immunity. Early innate responses are especially important since they greatly influence the subsequent responses that develop in the immune cascade. Such responses are believed to explain considerable differences between the salmonid species in susceptibility to lice [10]. Limited epithelial hyperplasia and inflammation after infection with the parasite were reported in Atlantic salmon (*Salmo salar L*.) and this was in contrast to highly resistant coho salmon (*Oncorhynchus kisutsch*) and chinook (*O. nerka*) salmon [11]. This can be related to inherent constraints of the immune system or its modulation by the parasite. In addition, Atlantic salmon possesses thin epidermal layer, sparsely distributed mucus cells, and exhibits low mucus lysozyme and protease activity as compared to other salmonids [12].

Knowledge of salmon immune responses to lice and their roles in protection against parasite is still limited. Until present, studies have addressed a relatively small number of immune parameters. Development of high-throughput analytical methods makes it possible to expand the search and to monitor large number of immune pathways in parallel at the gene expression level. In a previous study, we used a 1.8 k cDNA microarray (SFA2 or immunochip) to examine the local and systemic responses of Atlantic salmon to lice within the whole infection period [13]. This platform included a relatively small number of genes and the early responses were represented with only one time-point - 3 days post infection (dpi.). In this paper we report immune related responses during the first 15 dpi, divided in five time-points. This enabled us to see how the host is responding during the early infection period. In this study, we used the Atlantic salmon oligonucleotide platform discussed in detail in [14]. Gene expression profiling was done in skin and spleen and real-time RT-qPCR analyses were performed in these tissues, and also in the head kidney.

## Results

### Lice count and a summary of gene expression changes

**Figure 1:**
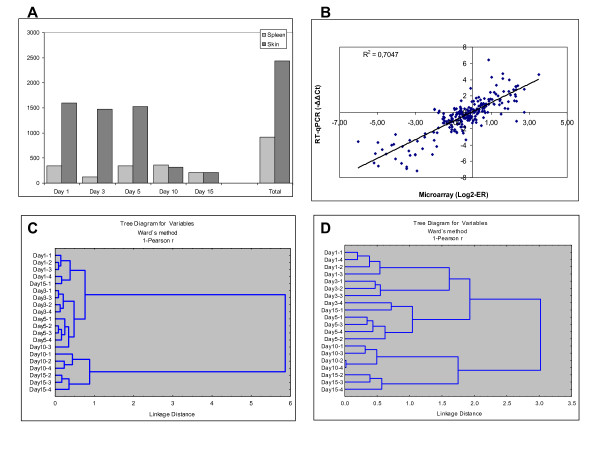
**An overview of transcriptomic responses to lice**. **A: **The number of differentially expressed features in skin and spleen (mean log_2_-Expression Ratio (ER) > |0.8|, p < 0.01, one sample t-test. **B**: Comparison of microarray and RT-qPCR results, pooled data for 18 genes analyzed in skin and spleen, n = 225. **C**, **D**: Hierarchical clustering of skin and spleen samples by expression profiles of DEG (Pearson r, Ward's method).

The number of lice was determined at 15 dpi (the last day of experiment), and high counts (58.4 ± 9.48 lice per fish, all at chalimus I to III stage) from 100 copepodids per fish of initial infection confirmed the lack of Atlantic salmon's ability to clear the parasite. However, the microarray analyses suggested rapid and sizeable transcriptomic responses to lice. The total number of differentially expressed features was 2438 in skin and 922 in spleen (Figure 1A and Additional file 1). Given low redundancy of the platform, these numbers are close to numbers of differentially expressed genes (DEG). While the magnitude of responses remained relatively stable within the whole study period in the spleen, the number of genes with expression changes in skin decreased markedly after 5 dpi. For validation of microarray results, genes that covered the whole range of expression ratios were chosen, and RT-qPCR analyses were performed in the same individuals (Figure 1B). The results of two independent methods were in good concordance: coefficients of linear regression and correlation (Pearson r) were equal to 0.84 and 0.80 respectively (complete RT-qPCR results are in Additional file 2).

**Figure 2:**
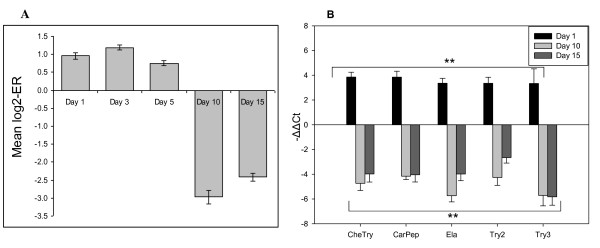
**Expression changes of proteases in spleen**. **A**: Microarray data are mean log_2_-ER ± SE for 24 genes with highly coordinated expression profiles. The lists of genes and accession numbers are in Additional file 3. **B: **RT-qPCR analyses performed with SYBR Green assays. Data are mean -ΔΔCt ± SE. Significant differences from control (n = 4, P < 0.01) are indicated with**. Note significant differences from control of all genes at all time points. CheTry, Chemytrypsin; CarPep, Carboxypeptidase; Ela, Elastase; Try2, trypsin-2; Try3, trypsin-3.

Hierarchical clustering suggested high consistency of the gene expression changes (Figure 1C, D). The samples (biological replicates) were grouped by the time-points with exclusion of one outlier (D15-1), which deviated from the common trend in both analyzed tissues. The samples from spleen and skin were divided in two large clusters (days 1-5 and days 10-15), which were sharply separated, especially in the skin. This suggested a bi-phasic response to lice and the K-mean clustering confirmed abrupt expression changes in a major part of genes between days 5 and 10 (data not shown). A notable example of bi-phasic regulation is a group of splenic proteases (trypsins and chemotrypsins, carbopeptidases and carboxylic ester hydrolases, elastase, proteinase E and choriolytic enzyme) and proteins involved in regulation of exocytosis (syncollin and endoplasmic reticulum protein ERp27). The microarray results were confirmed with RT-qPCR (Figure 2 and Additional file 3).

Search for the enriched functional classes and pathways in the present microarray data illustrate the thematic associations of gene expression changes. By functions of DEG, responses to lice were much more diverse and complex in skin, which was the target site for the parasite (Table 1). The changes were associated with cell maintenance (metabolism of amino acids and sugars, mitochondrion and cytoskeleton (including motor proteins), protein biosynthesis, modification and transport, regulation of redox status, DNA replication and repair), cell communication and reparation of tissues. By result of statistical analysis, enrichment was greatest in classes related to basic metabolic functions (mitochondrion, glycolysis and ribosomes). The immune functional groups comprised a relatively small fraction of changes in the skin (only two KEGG pathways) but were predominant in the spleen (five of ten terms included in Table 1); inflammatory response and complement and coagulation cascades were the most enriched terms. The study focused on the immune responses and therefore in presentation of results preference is given to genes with known immune roles.

**Table 1:** Enrichment of GO classes and KEGG pathways in the lists of DEG

Functional group, pathway	Features^1^	p-value^2^	Vocabulary
***Skin***			
Mitochondrion	177 / 1104	0.000	GO
Glycolysis / Gluconeogenesis	33 / 117	0.000	KEGG
Pentose phosphate pathway	13 / 48	0.009	KEGG
Glutamate metabolism	16 / 48	0.000	KEGG
Glutathione metabolism	13 / 49	0.011	KEGG
Ribosome	47 / 172	0.000	GO
Protein folding	33 / 178	0.015	GO
Protein modification	44 / 246	0.009	GO
Protein transport	78 / 473	0.004	GO
Cytoskeleton	89 / 579	0.013	GO
Myosin complex	23 / 86	0.000	GO
Endoplasmic reticulum	140 / 992	0.027	GO
Cell redox homeostasis	11 / 43	0.027	GO
Double-strand break repair	7 / 24	0.050	GO
Anti-apoptosis	41 / 200	0.001	GO
Positive regulation of apoptosis	12 / 51	0.037	GO
**Antigen processing and presentation^3^**	17 / 69	0.007	KEGG
**Leukocyte transendothelial migration**	32 / 186	0.043	KEGG
Cell adhesion	83 / 545	0.020	GO
Tight junction	38 / 208	0.011	KEGG
Heparin binding	18 / 90	0.043	GO
Keratinization	8 / 21	0.006	GO
TGF-beta signaling pathway	24 / 113	0.008	KEGG
***Spleen***			
**Inflammatory response**	22 / 213	0.000	GO
**Complement and coagulation cascades**	29 / 85	0.000	KEGG
Peptidase activity	23 / 145	0.000	GO
**Acute-phase response**	8 / 20	0.000	GO
**Chemotaxis**	10 / 81	0.003	GO
Basement membrane	10 / 68	0.000	GO
**Cell adhesion**	36 / 545	0.020	GO
Extracellular space	47 / 375	0.000	GO
Heparin binding	13 / 90	0.000	GO
Neuroactive ligand-receptor interaction	14 / 173	0.036	KEGG

^1 ^Numbers of genes among DEG and on the microarray platform. ^2^Yates' corrected chi-square. ^3^Immune related groups and pathways are highlighted with bold.

### Humoral immunity and inflammation

**Figure 3:**
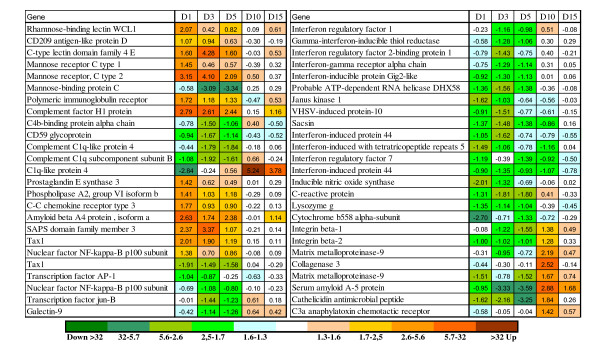
**Examples of immune genes with differential expression in skin (microarray results)**. Data are mean log_2_-ER (n = 4).

**Figure 4:**
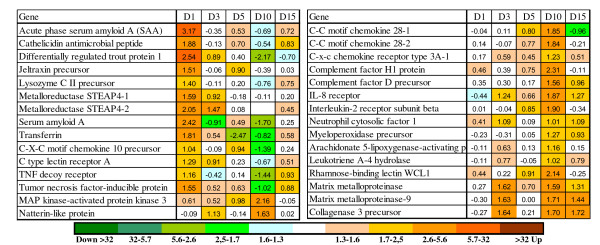
**Examples of immune genes with differential expression in spleen (microarray results)**. Data are mean log_2_-ER (n = 4).

**Figure 5:**
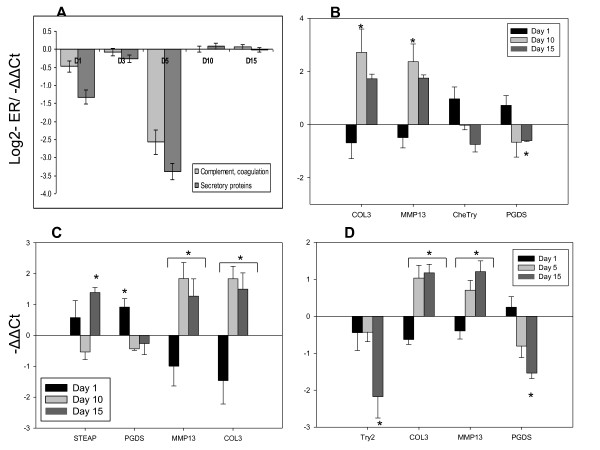
**Temporal expression changes**. **A**: plasma proteins in spleen (microarray results). Data are mean log_2_-ER ± SE for 26 genes (complement and coagulation cascade) and 55 genes (secretory proteins) with similar expression profiles. The lists of genes and accession numbers are in Additional file 4. **B-D: **RT-qPCR analyses performed with SYBR Green based assays in skin **(B)**, spleen **(C)**, and head kidney **(D)**. Data are mean -ΔΔCt ± SE. Significant differences from control (n = 4, P < 0.05) are indicated with *. COL3, Collagenase 3 precursor; MMP13, Matrix metalloproteinase-13; CheTry, Chemytrypsin; PGDS, Prostaglandin D synthase; STEAP, Metalloreductase STEAP4; Try2, trypsin 2.

Rapid responses to the parasite and transmission of signal from the damaged sites to the internal organs were confirmed with up-regulation of pro-inflammatory genes in both skin and spleen. The complement system is part of both innate and adaptive immune system, and plays a major role in recognition and elimination of pathogens. Several lectins with early (1 dpi) induction in skin (Figure 3) have unknown roles but may be needed for detection of pathogen; the calcium dependent (C-type) lectin domain family 4 E is expressed in macrophages and other Ag presenting cells [15]. In theory, lectins can activate one of the complement pathways. In this respect, it is noteworthy to mention down-regulation of several genes for C1Q-like proteins that can trigger the classical pathway, which could mean preferential activation of the lectin pathway. Decreased expression was shown for two negative regulators of complement: CD59 and C4b-binding protein. Phospholipase A2 and prostaglandin E synthase 3 are involved in biosynthesis of inflammatory regulators and several more immune effectors showed rapid up-regulation. The RT-qPCR analyses of IL-1B, IL-12, TNF-α did not find significant expression changes in skin, spleen, and head kidney (see Additional file 2). The components of the NFKB pathway changed expression in both directions while a panel of IFN-dependent proteins were down-regulated; many of these have shown strong responses to viruses [14]. Given large distance between the spleen and the skin, we could anticipate preferential regulation of genes for proteins exported to plasma and body fluids including acute phase proteins (serum amyloids, lysozyme C and transferrin) (Figure 4). Several lesser known proteins have been attributed to this functional group; these are jeltraxin, which is similar to C-reactive P component and serum amyloid P component, differentially regulated trout protein 1 [16] and LPS neutralizing protein cathelicidin. In addition, rapid up-regulation was observed in a number of possible pro-inflammatory genes including several TNF-dependent genes (TNF decoy receptor, metalloreductase STEAP4 and TSG6). In parallel, a large group of genes for plasma proteins decreased expression: highly coordinated changes were seen in the components of complement and coagulation cascade (26 genes) and in a diverse group of 55 genes that among other included apolipoproteins and glycoproteins, macroglobulins and protease inhibitors, proteins binding copper, iron and heme, scavengers, chemokines and cytokines (Figure 5A).

The temporal patterns of inflammatory changes were different in the analyzed tissues. In skin, many genes had similar expression profiles during 1-5 dpi, while in spleen the acute phase proteins showed a short-term increase only at 1 dpi. However in both skin and spleen the character of innate immune responses changed dramatically after 5 dpi. The switch of transcriptomic program was marked with abrupt temporary down-regulation of splenic plasma proteins, which was similar to that observed at 1 dpi but with greater magnitude (Figure 5A). A hallmark of the second phase was up-regulation of several matrix metalloproteinases: MMP9 (gelatinase) and MMP13 (collagenase 3), which was observed in skin, spleen and head kidney - the latter was analyzed with RT-qPCR (Figure 5B-D). These inducible enzymes have a wide range of roles, from massive degradation of extracellular matrix and tissue remodeling to limited proteolysis and subtle regulation of immune processes [17,18]. Various pro-inflammatory genes including chemokines and effectors showed up-regulation after 5 dpi.

### Cellular responses, acquired immunity

**Figure 6:**
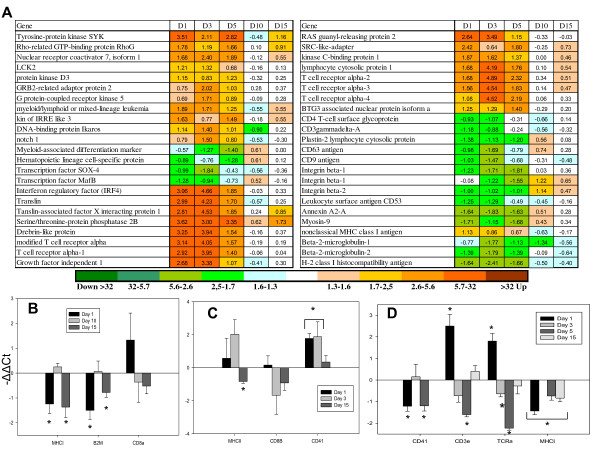
**Examples of genes involved in cellular immunity with differential expression in skin and head kidney**. **A**: microarrays in skin, data are mean log_2_-ER (n = 4). The gene functions are explained in the text. **B-D**: RT-qPCR analyses of MHC and T-cell markers in skin (**B **&**C**), and in head kidney (**D**) performed with SYBR Green and TaqMan based assays. Data are mean -ΔΔCt ± SE. Significant differences from control (n = 4, P < 0.05) are indicated with *.

The gene expression profiles in skin (Figure 6A) indicated rapid alterations of the composition of immune cells in the target site. Stable up-regulation during 1-5 dpi was observed in a panel of signal transducers: LCK2, protein kinase D3, RAS homologue member G (RhoG), spleen tyrosine kinase (SYK), GRB2-related adaptor protein 2, G protein-coupled receptor kinase 5, RAS guanyl-releasing protein 2, which are known for their important roles in regulating immune cell movement [19,20]. Several of these genes have shown preferential expression in salmon peripheral blood leukocytes in previous microarray study [14]; however, their association with specific cell lineages remains unknown. Microarray analyses showed decreased abundance of transcripts for proteins that have a major part in transendothelial migration of leukocytes, including annexin 2, myosin 9 - a non-muscle motor protein, and integrin beta; CD9 and CD63 expressed on leukocyte membranes interact with integrins and proteins of extracellular matrix. Down-regulated CD53 mediates activation of leukocytes and MafB is the myeloid associated differentiation marker. We did not see induction of myeloid-specific genes while a number of events suggested recruitment and activation of lymphoid cells. Increase was observed in a panel of T-cell-specific genes including T-cell receptor alpha (TCRα), serine/threonine-protein phosphatase 2B, L-plastin, drebrin suggesting preponderance of T lymphocytes among immune cells that appeared in the target sites (Figure 6A). Up-regulation of TCRα and CD3ε in the head kidney at 1 dpi and decrease at 5 dpi (Figure 6D) implied rapid recruitment of T-cells from this depot. The nature of these cells remains unknown. No expression changes of CD8 were detected though the microarray platform included probes to alpha and beta chains whose performance was confirmed in studies with viral diseases including cardiomyopathy syndrome (CMS), heart and skeletal muscle inflammation (HSMI), and the infectious salmon anemia (ISA) (unpublished results). The RT-qPCR analyses found a short-term up-regulation of CD4-1 in skin (Figure 6C). An interesting finding was expression changes of genes that control differentiation of lymphocytes. This was shown by an increase in several genes that regulate early lymphopoiesis, such as kin of IRRE like 3, myeloid/lymphoid or mixed-lineage leukemia, notch 1, Ikaros, growth factor independent (Figure 6A). Translin and translin-associated X interacting protein 1 are required for somatic recombination of genes encoding immunoglobulins (Ig) and T-cell receptors [21], while BTG3 and IRF4 stimulate their transcription. This may mean that terminal differentiation of T-cells takes place in the infected sites and we came to a similar conclusion in our studies of a viral disease CMS (unpublished data).

**Figure 7:**
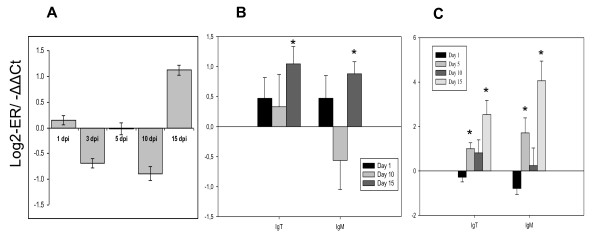
**Expression of immunoglobulins**. **A**: microarray analyses in spleen. Data are log_2_-ER ± SE for 28 independent probes. **B, C**: RT-qPCR analyses in spleen (**B**), and skin **(C) **performed with SYBR Green and TaqMan based assays. Data are mean -ΔΔCt ± SE. Significant differences from control (n = 4, P < 0.05) are indicated with *.

We did not find any indications of T-cell mediated immunity in subsequent responses. Down-regulation of MHCI, B2M in skin and head kidney during 1-5, and 15 dpi, and MHCII in skin 15 dpi (Figure 6), suggested absence of antigen presentation to T-cells. After 5 dpi the T-cell related genes showed no expression changes in skin. In contrast, there was evidence for the development of B cell mediated immunity. Despite an early regulation in skin of polymeric immunoglobulin receptor (pIgR), a key molecule in transcytosis of Igs (Figure 3), neither microarray nor RT-qPCR analyses showed early regulation of Ig genes in skin. Rapid (1 dpi) up-regulation of IgM and IgT in the head kidney followed with decrease at 5 dpi (additional file 2) suggested recruitment of B cells. However since no increase of B cell-specific transcripts were detected in skin at 1-3 dpi, they probably did not appear in the target site. However, RT-qPCR analyses revealed gradual increase of IgM and IgT transcripts from 10 to 15 dpi (Figure 7). Up-regulation of several isoforms of lymphocyte G0/G1 switch protein 2 at 5 dpi (data not shown) probably marked an onset of adaptive immune responses in the spleen. A large panel of Ig transcripts showed decrease at 10 dpi followed with up regulation at 15 dpi.

## Discussion

**Figure 8:**
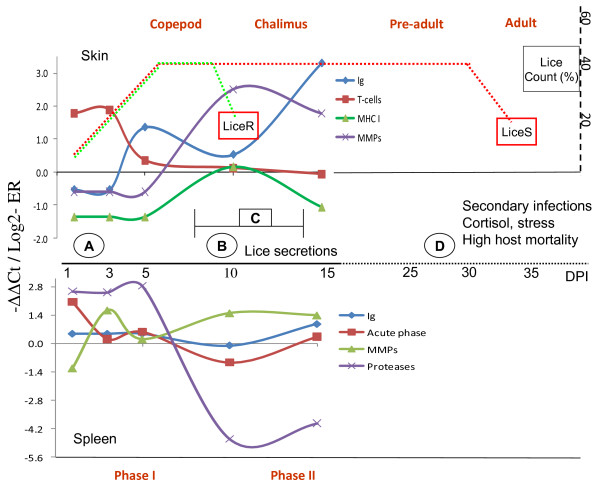
**Summary of salmon responses to lice in skin and spleen**. The present study dealt with responses until 15 dpi as shown in the left side of the figure. Host responses to mature lice stages from literature are shown by a dotted line (right side). The figure illustrates the bi-phasic responses to *L. salmonis *with abrupt changes in gene expression profiles taking place between 5 and 10 dpi, when lice molts from copepodids to chalimus. Ig genes show an initial decrease followed by gradual increase. MHC and related genes are down regulated. **A**: early sensing was witnessed both in skin and spleen. At point **B**, PGE_2 _and other secreted components start to increase [31,34] and this may lead to immune modulation, and can partly explain the pronounced shift in immune responses. Resistant salmonids (e.g. coho salmon) reject lice at point **C**, 7-14 dpi [10,11] and the number of lice per fish (LiceR) starts to decrease (see the hypothetical curve on the right side). In susceptible species including Atlantic salmon, the lice number (LiceS) remains relatively stable until they reach the pre-adult/adult stages where lice falls off the fish possibly due to aging, competition, and other factors. In spleen, secretory proteases show dramatic fluctuation. Increased MMPs in all tissues suggest inflammation. When the louse enters the pre-adult stage (i. e, ~ after day 20), the effects on host increase and can cause high mortality. The risk of secondary infections increase, and cortisol levels rise significantly (point **D**), which indicates severe stress [9,26,34]. At this point, it is difficult to differentiate the direct and stress mediated effects of lice [26]. The figure summarizes responses observed after single pulse infection. However, under natural conditions, salmon can carry parasites at different developmental stages and responses can be modified with diverse environmental factors.

The Atlantic salmon is highly susceptible to *L. salmonis *and the present study was thus designed to identify host responses due to the early infectious stages from 1 to 15 dpi. After settlement, copepodids spend 7 to 11 days (at 9°C) on the host before all have completed the molt and are physically attached to the host by a frontal filament. We focused on the first 15 days after infection and the host responses were related to copepodids (day 1 to 5), mixed copepodid and chalimus (day 10) and chalimus (day 15) (Figure 8). We used advantages of multiple gene expression profiling with a 21 k oligonucleotide microarray, which covers the major fraction of protein-coding genes in Atlantic salmon. Transcriptional responses to salmon louse were analyzed in skin, the first entry point for the parasite, and in the spleen. The latter was selected due to the important role as a lymphoid organ [22] and furthermore, in our previous microarray study [13] we found greater gene expression changes in salmon spleen in comparison with the head kidney, another major immune organ of teleost fish. Our data revealed a strong host response one day post infection with a pronounced switch in gene expression pattern taking place between 5 and 10 dpi. This switch corresponds to the period where lice molt from copepodids to chalimus I ending up with a new transcription pattern at day 15 where all surviving parasites have developed into chalimus (Figure 8). In contrast to Pacific salmon, Atlantic salmon show limited tissue response to *L. salmonis *infections [10]. The reason for this is unknown but a recent study [23] indicates that the Pacific and Atlantic form of *L. salmonis *may represent two different species and this may account for some of the differences. In addition to immune parameters, differing resistance of salmonids to *L. salmonis *can be related to the structure of skin, composition of mucus, and environmental factors [5,9-12,24].

Little is known about how teleosts respond to parasites in general and to the salmon louse in particular. By using a microarray approach it is possible to screen a large number of markers and to identify both known and novel host responses to the pathogen of interest. Use of genomic tools allowed reconsidering of views based on the studies with limited sets of immune parameters. It was thought that louse do not cause significant effect in Atlantic salmon at early stages [25]. Weak inflammation at the site of attachment was regarded as a plausible explanation of higher susceptibility of Atlantic salmon in comparison with closely related species, such as sockeye and coho salmon (reviewed in [26]). However, transcriptomic analyses did not show low levels of immune responses to lice in Atlantic salmon. Dramatic gene expression changes were seen immediately after infection in the target site (skin) and in the spleen; both local and systemic sensing was rapid and large by scale. Given that most differentially expressed genes are not those that are commonly included in studies of salmon immunity, it would be difficult to detect these changes based on the candidate genes approach. An unexpected finding was involvement of splenic proteases. Dramatic expression changes of a group of genes encoding functionally related proteins imply their important role, which remains completely unknown. Thus, results of transcriptome analyses suggest that low resistance of Atlantic salmon to lice appears to be accounted for by the character of immune response rather than the scale of the response. The results elucidated immune processes that are activated but most likely do not confer substantial protection against the parasite.

High-throughput analyses revealed a bi-phasic response to lice. Modulation of responses by the parasite can be considered as one possible explanation. It is well documented that parasites have the ability to modulate host response to avoid rejection by the host and by this increase survival. For ectoparasites this can be conducted by releasing excreted products to the host surface or the site of feeding. Based on knowledge from other ectoparasites it is likely that *L. salmonis *releases a diversity of secretory/excretory products when it settles on a suitable host. Recently it was shown that horse fly (*Tanabus yao*) release a wide diversity of molecules when feeding and these molecules where shown to affect a range of biochemical and physiological processes in the host [27]. Salivary gland extracts from ticks suppress lymphocyte proliferation and cytokine response [28]. Parasites such as *Leishmania *utilize a number of immune avoidance strategies [29], some of which resembling that of tumor cells [30]. Immune suppression by lice has been reported in several publications. Salmon louse releases molecules that affect host response [31] and a few of these have been identified [31,32]. Lice produce PGE2, trypsin-like proteases, and other products that suppress the immune system of Atlantic salmon [33]. Significant reduction of oxidative and phagocytic activities of macrophages [25], and reduced transcription of IL-1β and COX-2 in lice infected salmon has been reported [31,34]. In the present study, a panel of pro-inflammatory cytokines analyzed with RT-qPCR (IL1-β, IL1R1, TNFα and IL-12) did not show significant response to lice. Furthermore, our findings indicated down-regulation of Ag presentation after infection with salmon lice, possibly affecting the conventional T-cell mediated adaptive immune response. Similar down-regulation of genes involved in Ag processing has been documented in Atlantic salmon infected by the protozoan ectoparasite that causes amoebic gill disease (AGD) [35]. This is interesting because *L. salmonis *is also implicated as a possible risk factor for AGD [36]. In similar host-parasite interaction studies, MHC II gene expression decreased in head kidney and skin after infection of carp with *Trypanoplasma borreli *[37], and rainbow trout with *Gyrodactylus derjavini *[38]. Besides, our microarray data shows down-regulation of lysosomal proteases (cathepsins), which process exogenous antigens for presentation by MHC II [39,40].

The character of inflammation changed during the copepodid-chalimus transition as well. Commonly acute and chronic inflammation is associated with cells of myeloid origin and lymphocytes, respectively. However, an opposite trend was observed in our study. As shown in Figure 6A and 6C, gene expression changes provided evidence for a rapid recruitment of T-cells in the damaged sites, indicating a short term T-cell mediated response early during infection (1-5 dpi), which completely disappeared after 5 dpi. It is worth noting that Atlantic salmon possesses diversified numbers T-cells and receptors [41,42]. Their functional roles remain undetermined, as the true cytokine profile of CD4 response is dependent on interactions between the pathogen and antigen-presenting-cells [43]. In mammals, natural T- cells expressing a conserved TCRα-chain can exhibit both CD4^+ ^and CD4^-^/8^- ^double-negative phenotype [44]. It is possible that in the present study, at least some of the lymphocyte responses could be MHC-independent, possibly belonging to unidentified lineages that are not associated with acquired immunity or immune memory either, as Atlantic salmon used in this study was not immunized previously against lice. These cells could be similar to innate T-like cells which function as natural killer T-cells recognising antigens presented by non-classical MHC molecules [45]. Microarray data showed up-regulation of non-classical MHCI molecules in the skin (Figure 6A). Induction of genes that control early stages of lymphocyte differentiation suggests involvement of precursor cells, which either resided in skin or were delivered with blood. Concurrent down-regulation of several genes in skin that control transendothelial migration indicates the depletion of leukocytes.

A hallmark of transit from acute to chronic inflammation was the systemic increase of MMP9 (gelatinase) and MMP13 (collagenase), which did not show expression changes during the first phase. Earlier we found preferential expression of these genes in salmon leukocytes [14]. The changes of transcript abundance could be due to either MMPs induction in activated resident immune cells (macrophages) or influx of leukocytes. The latter possibility is supported with simultaneous up-regulation of integrins and C3a anaphylatoxin chemotactic receptor in skin and neutrophil cytosolic factor in spleen (Figs 3 &4). The observed changes can be a consequence of chronic stress and increased production of cortisol. In this respect, it is noteworthy that Fast et al. [34] found no changes of plasma cortisol levels during the first 15 dpi in Atlantic salmon infected with *L. salmonis*, while its increase at 26-33 dpi was in parallel with the induction of pro-inflammatory mediators (IL-1β and TNFα). Overall, cortisol has an immune suppressive action. However, a remarkable feature of salmon MMPs is induction with both inflammatory stimuli [46] and stress [47]. Recently we observed up-regulation of MMPs in salmon with cortisol implants (manuscript under preparation). Previously we reported a sustained induction of MMPs as a characteristic feature of lice infection in Atlantic salmon [13]. MMP-9 in carp LPS stimulated leucocytes shows a bi-phasic profile: increase until 48 hours, decline, and another increase at 168 hours, indicating its role both in early inflammation and later stages of tissue remodelling [17].

Vaccines are discussed as a possible measure against salmon louse. Immunization of fish against *L. salmonis *may be facilitated by an improved understanding of the adaptive immune system and molecules involved therein, particularly how the host responds to parasites. One of the limitations with vaccine development could be the limited exposure of louse to blood and thereby serum antibodies (reviewed in [8]), and mucosal immunity might play a major role here as *L. salmonis *are colonizers of cutaneous mucosa of salmonids. Mucosal epithelial cells serve as an initial barrier and, in addition, they are involved in adaptive immunity by Ag presentation and production of Igs along with complement, lectins, CRP, lysozymes, proteolytic enzymes and other effectors [48-50]. Antibodies at the surface of skin mucus can block ectoparasites from infestation or reduce infestation success [50]. IgT/IgZ is a teleost specific antibody class first discovered in rainbow trout and zebrafish [51,52]. In salmon, there are three highly similar IgT sub-variants [53]. A recent study indicated that they might be differentially regulated [54]. IgT is associated with mucosal immunity, similarly to the mammalian IgA [55]. IgT transcription in rainbow trout gut was up-regulated more than 700 fold in fish that survived infection with the parasite *Ceratomyxa shasta *[55]. This immunoglobulin may coat gut luminal bacteria thus preventing their attachment and invasion of the gut epithelium [55]. While transcript levels of IgT and IgM in mucosal tissues of naïve Atlantic salmon are relatively low [53], we documented up to ten fold increase in skin after infection with *L. salmonis *(Figure 7C). An increase of IgT and IgM transcripts in skin and spleen may indicate an onset of adaptive immune responses at later stages of infection. It is worth mentioning here that microarray data has showed an early up-regulation of pIgR, a key molecule involved in the transport of Igs to mucosal surfaces [55-57]. However, we did not observe an early increase in Ig transcripts at the target site. In addition to transcytosis of Igs, pIgR has an important role in innate immune functions by attaching to host and pathogenic factors, as well as protecting Igs from proteolytic degradation [57,58].

## Conclusions

In this paper we studied gene expression changes in Atlantic salmon skin, spleen and head kidney during the first 15 days post infection by *L. salmonis*, using microarray and RT-qPCR (results are summarised in Figure 8). The findings clearly indicated early sensing at 1 dpi with induction of genes involved in innate immune reactions, including lectins and enzymes of eicosanoid metabolism in skin and acute phase proteins in spleen. This was followed by regulation of a diverse array of genes including MMPs and immunoglobulins. The responses are bi-phasic with large shift in transcript profiles of many genes during the time window corresponding to the copepod-chalimus transition. Gradual increase of Ig transcripts from 1-15 dpi in skin and spleen, possibly indicated mounting of adaptive immunity, which was supported by the up-regulation of putative lymphocyte G0/G1 switch proteins at 5 dpi in the spleen. The responses, however, did not result in appreciable level of protection, as revealed by the lice load on fish at the end of the study. Down-regulation of the antigen presenting MHCI and related molecules, and absence of T-cell induction at later stages suggested lack of T-cell dependent acquired immunity. Further biochemical and functional studies of immune mechanisms of IgT at mucosal sites in salmon, in the context of lice infection will greatly contribute to a better understanding of how adaptive immunity is orchestrated in salmon with regard to mucosal defences. Furthermore, the large group of secretory splenic proteases, which show the greatest transcriptional fluctuations (up- and down-regulation), deserve a closer attention.

## Methods

### Challenge experiment

**Figure 9:**
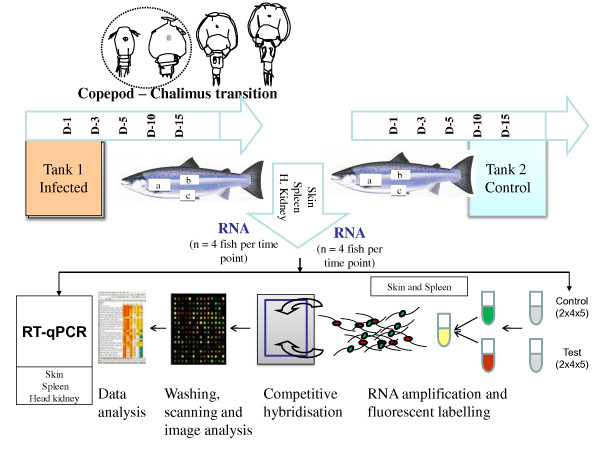
**Set-up of the experiment**. Atlantic salmon was challenged with *L. salmonis *at the copepod stage. Skin, spleen, and head kidney were sampled from challenged and control fish at 1, 3, 5 dpi (corresponding to the copepod stage); 10 and 15 dpi (chalimus stage). Boxes a, b, and c respectively indicate approximate positions on the fish where tissues (head kidney, skin, and spleen) were removed. A total of forty samples of spleen and skin form infected salmon (4 individuals from the 5 time points) were used for microarray analyses. Test samples were labeled with Cy5 and hybridized to pooled control samples labeled with Cy3 from the same time-points. Competitive hybridization to the arrays was followed by washing, scanning, image analysis, and data analysis. Selected genes were analyzed with RT-qPCR.

Atlantic salmon in the size range of 100-200 g, which has not previously been in contact with *L. salmonis*, were placed in two different tanks containing full salinity water (one for control and another for *L. salmonis *infection), at the Institute of Marine Research (IMR) in Bergen. A hatchery and culturing system that enables laboratory maintenance of salmon louse throughout its life-cycle was developed recently [59]. Salmon louse of LsGulen strain [59] were reared in the hatchery and egg strings were collected and placed into an incubator until they reached the copepodid stage. Atlantic salmon were challenged with *L. salmonis *(approximately 100 copepodids per fish). Commonly, approximately one third of the copepodids added to a tank are found on fish during the pre-adult/adult stages [59]. Following infection, tissues of skin, spleen, and head kidney were sampled 1, 3, 5 dpi (corresponding to the copepod stage); 10 and 15 dpi (chalimus stage). Control fish were sampled in a similar manner (Figure 9). Immediately after sampling, tissues were stored in liquid nitrogen and then transferred to -80°C freezer. The fish were kept at a temperature of 9 ± 1°C during the entire experimental period, and fed with commercial diet once daily. Louse load on each fish was counted at the end of the experiment, 15 dpi.

### RNA and cDNA preparation

RNA was isolated using the iPrep™ TRIzol^® ^Plus kit (Invitrogen), and purified with RNeasy mini kit (QIAGEN). The quantity and quality of the total RNA was assessed using a NanoDrop Spectrophotometer (NanoDrop Technologies) and an Agilent 2100 Bioanalyzer (Agilent Technologies). Samples with RNA integrity number (RIN) of 8 or higher were accepted for microarray analyses. cDNA was synthesized by reverse transcription of RNA using the qScript™ cDNA Synthesis Kit (Quanta BioSciences). Total RNA and cDNA were stored at -80 and -20°C respectively until use.

### Microarray analyses

RNA amplification and labelling were performed using Two-Colour Quick Amp Labelling Kit and Gene Expression Hybridization kit (for fragmentation of labeled cRNA) following the manufacturer's instructions for 4 × 44 k microarrays (Agilent Technologies). Nofima Marin's salmon oligonucleotide microarray SIQ3 (GEO GPL10706) was fabricated by Agilent Technologies and annotated with STARS bioinformatic package [14]. The features were assigned to the functional classes (GO) and pathways (KEGG). In addition, custom annotations were implemented based on literature and other public sources. Four biological replicates in each of 5 time-points per tissue (skin and spleen), which comprise a total of 40 microarrays (one array per sample), were included in the analyses (Figure 9). The input of total RNA used in each reaction was 500 ng. Pooled control samples were prepared by mixing equal RNA concentrations from each individual sample of control fish per each time point. Individual samples of infected fish were labeled with Cyanine 5 (Cy5), while control samples were labeled with Cy3. Following labeling, amplification, purification, and quantification, 825 ng of both the Cy5-labeled test cRNA samples and Cy3-labelled control samples were mixed and hybridized (competitively) to the arrays (Figure 9). Over night hybridization (17-hours, 65°C, and rotation speed of 10 rpm) was performed in hybridization oven (Agilent Technologies). After hybridization, arrays were washed with Gene Expression Wash Buffers 1 and 2 and scanned with a GenePix 4100A (Molecular Devices, Sunnyvale, CA, USA). GenePix Pro 6.0 was used for spot to grid alignment, feature extraction and quantification. Assessment of spot quality was done with aid of GenePix. After filtration of flagged low quality spots, Lowess normalization of log_2_-expression ratios (ER) was performed. The differentially expressed genes (DEG) were selected by criteria: log_2_ER > |0.8| and p < 0.01, (sample t-test, null hypothesis log_2_ER = 0) in at least one time-point. The microarray data were submitted to GEO, GSE26981 (skin) and GSE26984 (spleen).

### RT-qPCR

Eighteen differentially expressed genes from the microarray experiment, covering the entire dynamic range of expression, were selected for verification with RT-qPCR. Preference was given to immune genes and to genes with unknown roles that showed strong responses to the parasite. In addition, T-cell markers, immunoglobulins (Ig), and genes of antigen presentation, and pro-inflammatory cytokines were included. RT-qPCR analyses were carried out with skin, spleen and head kidney. TaqMan^® ^probe, and SYBR Green based Real-Time PCR assays were designed using Primer express 3.0 (Applied Biosystems), and Beacon Designer 7.8 (PREMIER Biosoft, USA) respectively (Table 2). When possible, primers or probes were designed to span between two exons. Fast SYBR^® ^Green Master Mix based PCR was performed using 7500 Fast Real-Time PCR System (Applied Biosystems). The PCR reaction mix contained 5 μl of Fast SYBR^® ^Green Master Mix (2 ×), 500 nM of each primer, and 2 μl of cDNA in a final volume of 10 μl. Thermal cycling was carried out according to the manufacturers protocol (Applied Biosystems) as follows: enzyme activation at 95°C for 20 sec, followed by 40 cycles of denaturation (95°C for 3 sec), and annealing/extension (60°C for 30 sec). In addition, Taqman probe based quantitative PCR was performed on a few selected genes using the 7500 Fast Real-Time PCR System (Applied Biosystems), and quantification of mRNA was performed in a single step assay (both RT and PCR steps carried out in the same tube) according to the Verso™ 1-step QRT-PCR low ROX kit (Thermo Scientific). PCR reaction mix containing 1-step qPCR low ROX mix (2 ×), enzyme mix, RT enhancer, 900 nM of each primer, 200 nM of TaqMan probe was mixed with 2 μl (50-100 ng) of RNA in a final volume of 12.5 μl. This is followed by thermal cycling steps of cDNA synthesis at 50°C for 15 min, an enzyme activation step of 95°C for 15 min, and 45 cycles of 95°C for 15 sec (denaturation) and 60°C for 60 sec (annealing/extension). All samples were run in duplicates (or triplicates) with non-template controls and non-reverse transcription controls on the same plate. Amplification of genomic DNA was checked by a melting curve analysis, which resulted in a single peak, indicating single product amplification. Elongation factor 1 alpha (EF1A) was used as an internal reference gene, as it has been shown to be the best reference gene for RT-qPCR studies in Atlantic salmon tissues [60]. We also have recent experience with samples similar to those analysed in this study and EF1A has shown an appropriate stability [53]. This transcript has also been represented in the microarray and did not show response to lice. To calculate ΔCt, the respective reference Ct values were subtracted from target Ct values of the control and test samples; and -ΔΔCt was calculated as: -ΔΔCt = - (ΔCt_Test _- ΔCt_Control_). PCR efficiencies were in acceptable range (Additional file 2). Statistical analysis of the RT-qPCR data was done using Microsoft excel and SigmaPlot 11.0. The data were presented as mean -ΔΔCt ± SE. Differences between control and test at each sampling point were assessed with Student's t-test.

**Table 2:** Primer/ probe list

S.N	Sequence Definition	Code		Primer/ probe (5'-3')	Product length	GenBank Accession (GI)
1.	Apoptosis regulator Bcl-X	Apo	Fwd	TACTAAGTGTTGCCGTTGA	109	209734267
			Rev	TAATCCAATCTGTGTCATTCTG		
2.	Beta-2-microglobulin precursor	B2M	Fwd	CACAGACAGACACAGACA	102	221220497
			Rev	CAACGATTGACAGAATAGACTT		
3.	Carboxypeptidase A1 precursor	CarPep	Fwd	AGCATACCAAGGACAACAC	75	209732661
			Rev	TACAACAGTACAATGACACAGT		
4.	Chymotrypsin B	ChyTry	Fwd	CTGTCCATACTGTATATTGCTAT	111	209734305
			Rev	GCTATAATGCTTAGGTGTTGTA		
5.	Collagenase 3 precursor	COL3	Fwd	ATCTGTGCTTACTACTAATCAAC	81	209156091
			Rev	GGGCTTCATCTTCTTTACTG		
6.	Complement C1q subcomponent subunit B precursor	C1qB	Fwd	CTGTCTGTCGTTGTCTTC	89	223649475
			Rev	ATGGTCTGTTGGTCTGTA		
7.	Elastase-1	Ela	Fwd	ACCGTCAACAAAGTCTTCA	75	S31963336
		Rev		CAGCAGAGCGATGTCATA		
8.	Fish virus induced TRIM protein	TRIM	Fwd	GCATGGCACAATAATAACT	75	S35697379
			Rev	GTCCAGATACACTCCTAC		
9.	Trypsin-2	Try2	Fwd	CAGTTGTCGGTTGAGATG	81	S18845530
			Rev	CAAGATGTGCCAGATAGC		
10	Trypsin-3	Try3	Fwd	CATTATTCTTCTCGCTCTG	81	S31964271
			Rev	TCATACCCTCCAACAATC		
11	Tumor necrosis factor alpha	TNFα	Fwd	ACAAAGAGGGCCAGGGATTC	100	126507266
			Rev	GAGGCCTGGCTGTAGACGAA		
12	Interleukin-1 receptor-like protein	IL1R1	Fwd	AGCAGGATGTCCTCGGTCTA	202	185136290
			Rev	TGGGTAGCGGTGTAGTTTCC		
13	Interleukin-1 beta	IL1β	Fwd	ACCGAGTTCAAGGACAAGGA	196	186288127
			Rev	GCAGCTCCATAGCCTCACTC		
14	Gamma-interferon-inducible lysosomal thiol reductase	GIILTR	Fwd	CTATGTGCCTTGGATTGT	79	209732609
			Rev	CAGAGTGAAGAGTGAAGAC		
15	HSP70_ONCMY Heat shock cognate 70 kDa protein	HSP70	Fwd	TCACTAGAGTCCTATGCT	85	CL7Contig1
			Rev	TTGTCTTGTCCTCATCAC		
16	Lipopolysaccharide-induced TNF-a factor homolog	LPSi-TNFα	Fwd	CAATTCCTTCGACCTCAT	85	209734201
			Rev	GCTCTTCTCCATACTGTC		
17	Metalloreductase STEAP4	STEAP4	Fwd	CTCCAACTCTGAAGACTATT	103	S48396453
			Rev	GAGCACTGTCAATCAATG		
18	Myosin, heavy polypeptide 9, non-muscle	Myo	Fwd	GCAGTTGAGACTCTACAGTGGAA	75	CB498954
			Rev	ACAGCGTGTTGAGTGTGGTT		
19	Programmed cell death protein 2	PCDP	Fwd	GCATAGACAGCCACAATC	81	209735097
			Rev	GAGCGTAACAACCTGAAG		
20	Prostaglandin E synthase 3	PGES	Fwd	TCCAGCCAATGTCTTAGT	99	223672934
			Rev	AAGCACGGTATAACTGAAC		
21	P-selectin precursor	P-sel	Fwd	CTGGTGATTCTATTGATGAC	86	209154193
		Rev		TTGACGCTGTAGTTGTAT		
22	CD8α	CD8α	Fwd	CGTCTACAGCTGTGCATCAATCAA	118	185135177
			Rev	GGCTGTGGTCATTGGTGTAGTC		
23	Interleukin-12	IL-12	Fwd	TCTACCTACACGACATTGTCCAGCC	62	209736091
			Rev	ATCCATCACCTGGCACTTCATCC		
24	Prostaglandin D synthase	PGDS	Fwd	ATCCCAGGCCGCTTCAC	59	304376917
			Rev	ACACGCATGTCATTTTCATTGTT		
			Probe	TTCACCAGCCAGCGTT		
25	Matrix metalloproteinase-13	MMP13	Fwd	GCCAGCGGAGCAGGAA	56	213514499
			Rev	AGTCACCTGGAGGCCAAAGA		
			Probe	TCAGCGAGATGCAAAG		
26	T-cell receptor alpha	TCRα	Fwd	GACAGCTACTACAGCCAGGTT		209736003
			Rev	CAGAATGGTCAGGGATAGGAAGTT		
			Probe	ACACAGATGCAAAGATC		
27	CD3ε	CD3ε	Fwd	TCAGGGCTCGGAAGAAGTCT	68	185135943
			Rev	GCCACGGCCTGCTGA		
			Probe	CCAAAAACCCACTTCCC		
28	CD4-like protein, variant 1	CD4-1	Fwd	GAATCTGCCGCTGCAAAGAC	75	185135736
			Rev	AGGGATTCCGGTCTGTATGATATCT		
			Probe	CCCAAACCAAAAGGATTC		
29	CD8β	CD8β	Fwd	GGAGGCCAGGAGTTCTTCTC	70	185135192
			Rev	GGCTTGGGCTTCGTGACA		
			Probe	ACCCGGAGAAACTC		
30	MHC class I antigen	MHC-I	Fwd	CAACGCCACAGGCAGTCA	64	25573077
			Rev	CGGTACTCATTCTGAGCTGTGTTAC		
			Probe	CACCAAACTCAAGTGGG		
31	MHC class II antigen	MHC-II	Fwd	CTCACTGAGCCCATGGTGTAT	117	223672978
			Rev	GAGTCCTGCCAAGGCTAAGATG		
			Probe	CTGGGACCCGTCCCTG		
32	Immunoglobulin mu	IgM	Fwd	TGAGGAGAACTGTGGGCTACACT	69	2182101
			Rev	TGTTAATGACCACTGAATGTGCAT		
			Probe	CATCAGATGCAGGTCC		
33	Immunoglobulin tau	IgT	Fwd	CAACACTGACTGGAACAACAAGGT	97	260766539
			Rev	CGTCAGCGGTTCTGTTTTGGA		
			Probe	AGTACAGCTGTGTGGTGCA		
34	Elongation factor 1A	EF1A	Fwd	CCCCTCCAGGACGTTTACAAA	57	224587629
			Rev	CACACGGCCCACAGGTACA		
			Probe	ATCGGTGGTATTGGAAC		

## Competing interests

The authors declare that they have no competing interests.

## Authors' contributions

TMT contributed to the overall experimental design, conducted the challenge test, the RT-qPCR analysis, performed the microarray experiment, involved in data analysis, drafted and wrote the manuscript. AK analysed the microarray data, drafted and wrote the manuscript. SS performed the microarray experiment and contributed to the overall manuscript. SA developed computer programs and database for management of microarray results and data mining. IH designed the study and contributed to the overall manuscript. FN designed the study, was involved in the challenge experiment, and contributed to the overall manuscript. All authors read and approved the final manuscript.

## Supplementary Material

Additional file 1 **Differentially expressed genes, complete microarray results**. Click here for file

Additional file 2 **Complete RT-qPCR results and PCR efficiencies**. Click here for file

Additional file 3 **Proteases with differential expression in spleen, microarray results**. Click here for file

Additional file 4 **Secretory proteins, components of complement and coagulation cascade differential expression in spleen, microarray results**. Click here for file
